# Correlative Brillouin and Raman spectroscopy data acquired on single cells

**DOI:** 10.1016/j.dib.2020.105223

**Published:** 2020-02-05

**Authors:** Silvia Caponi, Sara Mattana, Maurizio Mattarelli, Martina Alunni Cardinali, Lorena Urbanelli, Krizia Sagini, Carla Emiliani, Daniele Fioretto

**Affiliations:** a Istituto Officina dei Materiali del CNR (CNR-IOM)—Unità di Perugia, University of Perugia, Perugia, I-06123, Italy; b Department of Physics, University of Florence, Via G. Sansone 1, 50019, Sesto Fiorentino, Italy; c Department of Physics and Geology, University of Perugia, Perugia, I-06123, Italy; d Department of Chemistry, Laboratory of Biochemistry and Molecular Biology, Biology and Biotechnology, University of Perugia, Via del Giochetto, Perugia, I-06123, Italy

**Keywords:** Biophotonics, Brillouin spectroscopy, Raman spectroscopy, Cell mechanics

## Abstract

The distribution of chemical species and the mechanical modulation inside a single cell or tissue are of fundamental importance to characterize their physiological activity or their pathological conditions [1–4]. Here we analyse these properties by means of label free, non invasive, spectroscopic methods. In particular, we use a recently developed micro-spectrometer, which acquires simultaneously Raman and Brillouin spectra on the same point with subcellular resolution [5]. The techniques ability to analyse the chemical composition and the mechanical properties of single cells has been tested on NIH/3T3 murine fibroblast cells grown in adhesion on silicon substrates. Here we report the data acquired from fixed cells after their oncogenic transformation. Mechanical and chemical evolution is evident by direct inspection of raw data. Sharing our experimental records can be valuable for researchers interested in the analysis of single cells by Raman and Brillouin spectroscopy in order: i) to compare data acquired by different set-ups and ii) to correctly model the fitting functions.

**Table:** Specifications Table

Subject	Biophysics
Specific subject area	Optical Brillouin and Raman spectroscopy
Type of data	Raw and graph. The raw data are available in "Appendix A. Supplementary data" of the present article as zip file.
How data were acquired	Custom build microscope coupled with Brillouin and Raman spectrometers [6,7]
Data format	Raw
Parameters for data collection	The laser beam with λ = 532 nm and power on the sample P<3.5 mW is focalized on the single cells using UPLSAPO 60XW Olympus objective with NA1.2. In order to control the position on the cell, the Petri dish, thermalized at 37 °C, was inserted in a dedicated sample environment placed on a xyz translator stage (PI 611-3S Nanocube XYZ). Thanks to the piezoelectric control, it reaches a spatial resolution of 10 nm in a motion range of 100 μm for each axis.
Description of data collection	The Raman spectra were acquired by RM- Horiba iHR320 Triax using a 600 grooves mm^−1^ grating and an N_2_ cooled CCD detector (1024 × 256 pixels). The acquisition of Raman spectra up to 3000 cm^−1^ frequency shift is possible in this condition. At the same time, the High Contrast HC version of the Sandercock type tandem Fabry Perot (TFP-2) interferometer was used to acquire Brillouin spectra.
Data source location	IOM-CNR c/o Department of Physics and Geology University of Perugia Italy
Data accessibility	With the article

**Table:** 

**Value of the Data** • The data here presented are acquired using the recently developed Brillouin-Raman micro-spectroscopy set-up [6] for the correlative analysis of chemical and mechanical properties of biomaterials at the microscale. The technique has been tested on the analysis of single cells. • The shared data can be used by researchers involved in the characterization of cells by Brillouin micro-spectroscopy. In particular these data can be useful if compared with the ones obtained using different experimental set-ups based on the VIPAs spectrometers [[8], [9], [10], [11], [12]]. • The high spectral quality due to the high resolution and high contrast of the experimental set-up, let to collect spectra where different fitting models can be tested. Moreover the evolution as a function of the position in an elastically heterogeneous system such single biological cell, can be used to test the technique ability to probe mechanical properties at the microscales [13].

## 1 Data description

**Fig. 1:**
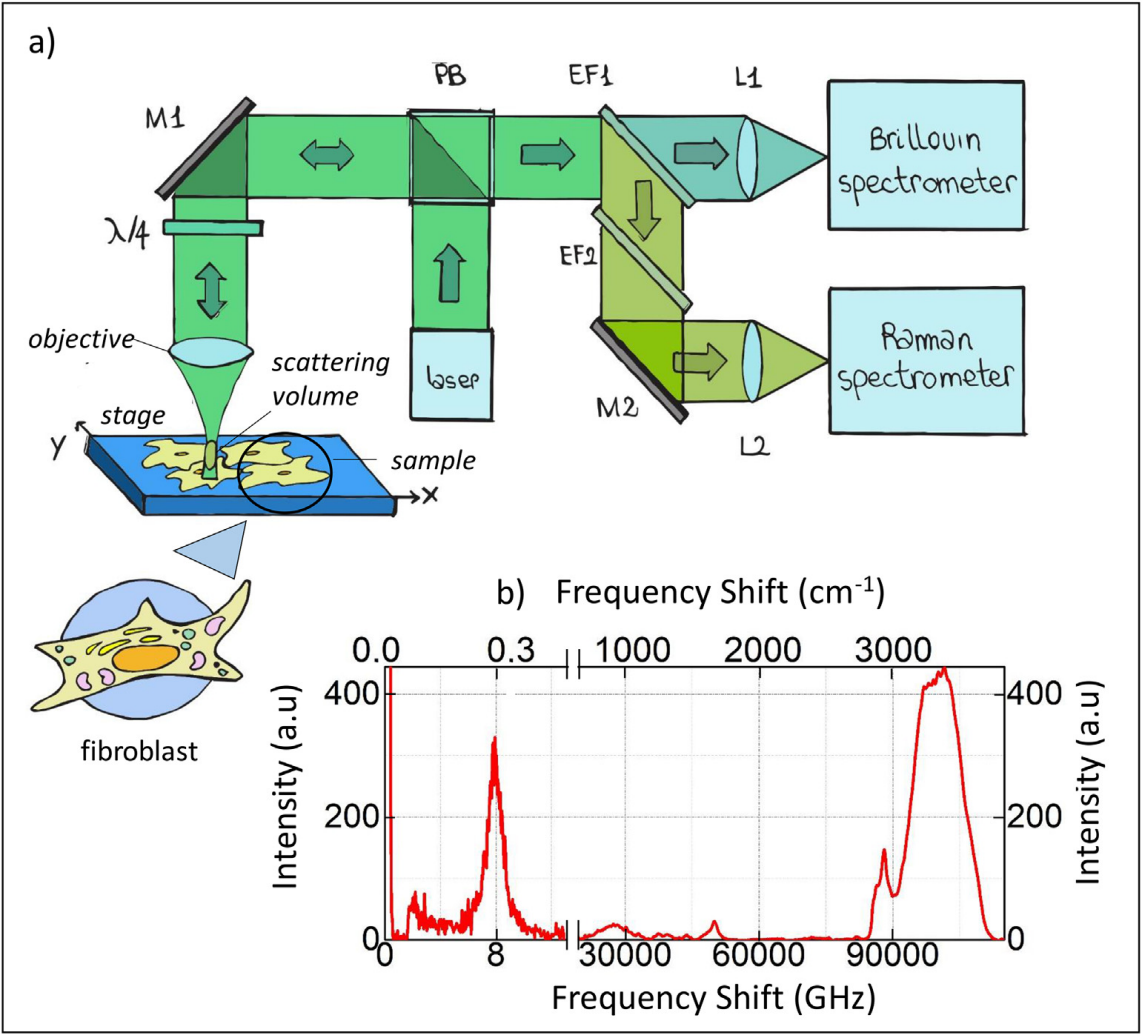
a) Schematic of the optical setup for simultaneous Brillouin-Raman micro spectroscopy. The optical components are labelled as: M (mirror), L (lent), EF (edge filter) and PB (polarized beam splitter) b) Brillouin and Raman spectrum simultaneously acquired inside the cell. For the Raman peaks assignment we refer to previous publications [5,14].

The growing importance to characterize mechanical properties, in addition to chemical ones, in biological framework is widely recognized in recent litterature [[1], [2], [3], [4]]. The data shared in the present paper were recorded investigating a single fixed cell using the novel experimental set-up recently assembled in our Lab able to simultaneously characterize chemical and mechanical properties of the investigated material [[5], [6], [7]]. The schematic picture of the optical system is reported in Fig. 1 a) (upper pannel). The Brillouin and Raman spectra simultaneously acquired probing the same position inside a single cell is reported in Fig. 1 b) (lower panel).

**Fig. 2:**
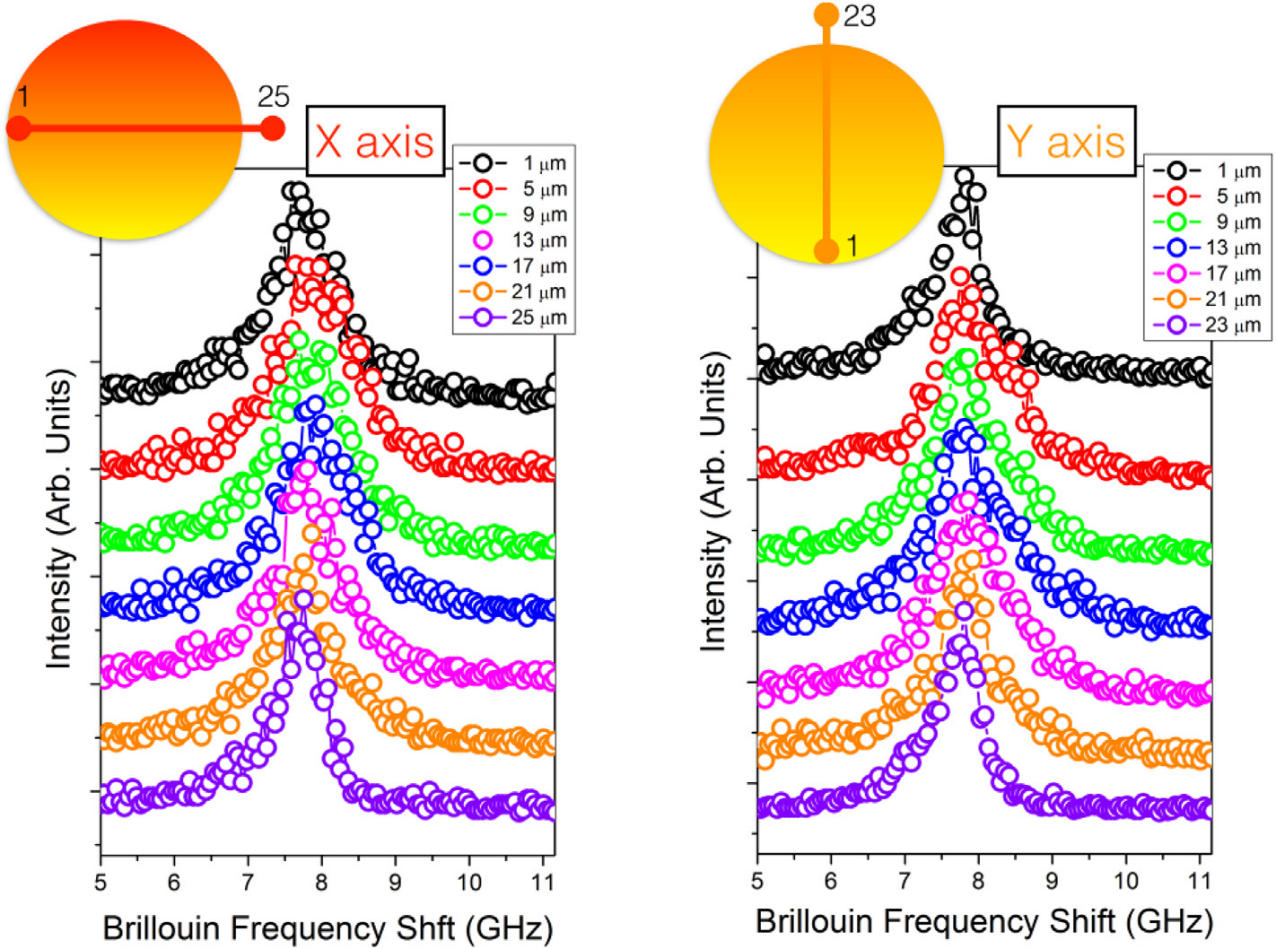
Sequence of Brillouin spectra probing different position inside the cell.

**Fig. 3:**
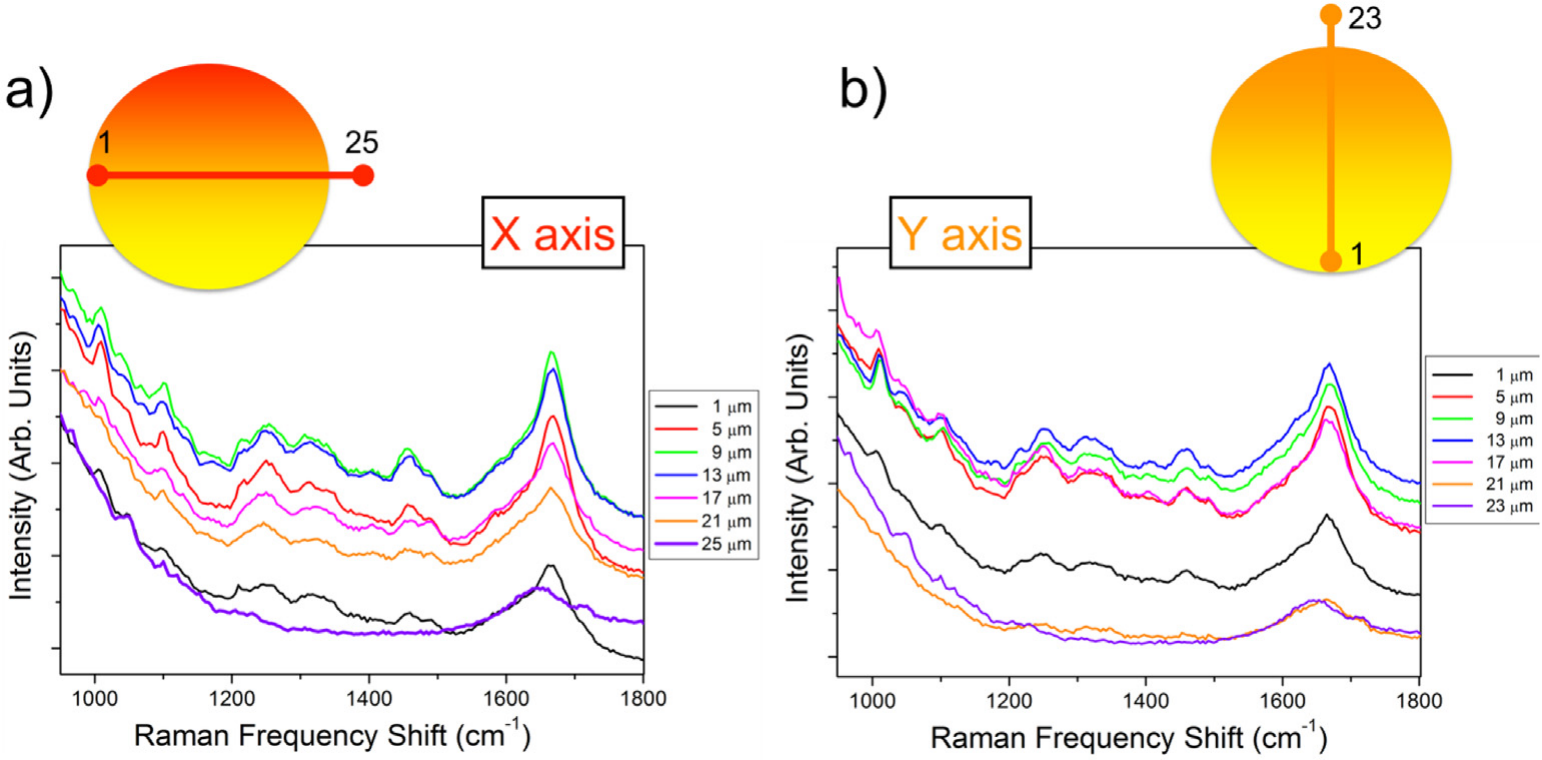
Sequence of selected Raman spectra probing the frequency region between 1000–1800 cm^−^^1^ acquired in different positions inside the cell.

**Fig. 4:**
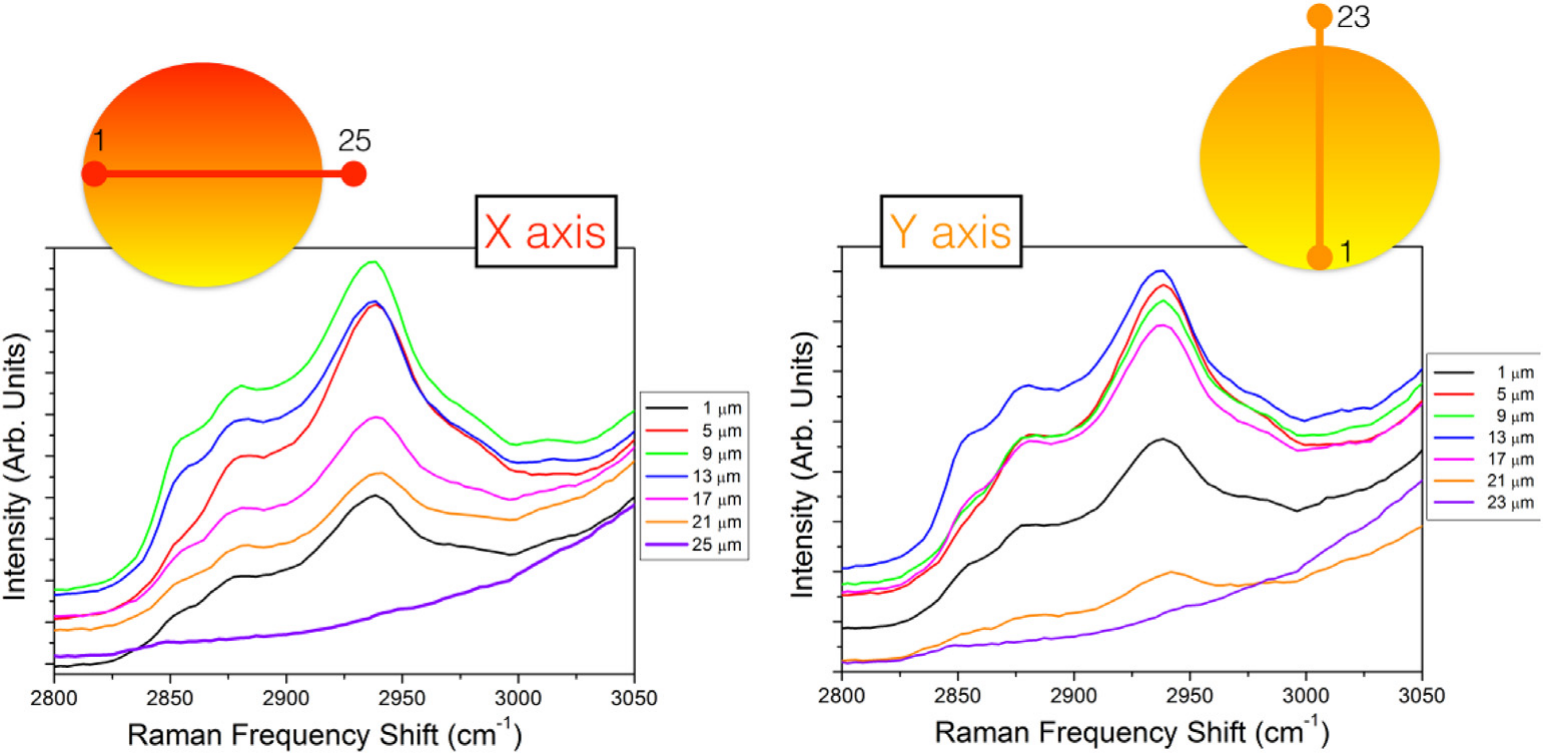
Evolution of CH_2_ and CH_3_ stretching Raman band probing different positions inside the cell.

The whole dataset is archived in BRaM_Dataset.zip present in "Appendix A. Supplementary data" of the present article. Both Raman and Brillouin data are simultaneously acquired probing the same point inside the cell. They were collected as a function of the position in a fixed and transfected NIH/3T3 murine fibroblast (see below for the details) grown in adhesion on silicon substrates. In particular, the data were collected moving with a step of 2 μm crossing the cell from one side to the other, entering from the plasmatic membrane, through the cytoplasm into the nucleus, and exiting from the other side. A selection of the collected Brillouin spectra are reported in Fig. 2 and the low and high frequency region of the Raman spectra are reported in Fig. 3 and Fig. 4 respectively. The right and the left panel of the figures show the measurements along two perpendicular directions within the cell (x and y axis). Directly from the raw data, it is possible to appreciate modifications in the spectral shape. In fact, moving through different cellular points, modulation in the elastic properties is evidenced by the shift and broadening of the Brillouin peak related to the emergence of high frequency component (for the detailed analysis see Ref. [5]). Moreover, modifications in the relative concentration of the different chemical species are visible by changes in the relative intensity of the Raman peaks present in both high and low frequency range of the spectra.

## 2 Experimental design, materials, and methods

The data were acquired using the Brillouin-Raman micro-spectroscopy set up extensively described elsewhere [5,6]. In brief, the laser light is focalized by a water immersion objective into the cell and the scattered light collected by the same objective is analysed in frequency by a HC-Tandem Fabry-Perot interferometer and by a single grating Raman spectrometer.

NIH/3T3 murine fibroblast cell line was purchased from American Type Culture Collection (ATCC). Cells were grown in Dulbecco Modified Eagle's Medium (DMEM) containing 10% (v/v) heat-inactivated fetal bovine serum (FBS), 100 U/mL penicillin, 100 U/mL streptomycin and maintained at 37 °C in a 5% CO _2_ humidified atmosphere. Cells were seeded in 6-well multiplates and transfected using Lipofectamine LTX with the expression vector pcDNA6/myc-His encoding the constitutively active mutant H-RasV12. The vector expressing the Ras mutant was previously described [15]. This mutation replaces the amino acid glycine with a valine, which makes the GTPase constitutively GTP bound. Transfected fibroblasts were selected using 4 μg/ml Blasticidin-S for 5 days. The expression of H-RasV12 was assessed by immunoblotting as previously shown [5]. Selected cells were trypsinized and seeded in silicon substrates sterilized with 100% ethanol washing and UV irradiation. Paraformaldehyde fixation was performed by incubating cells with 4% paraformaldehyde in PBS for 10 minutes at room temperature, then cells were washed twice with PBS. For the spectroscopic measurements, the cells were immersed in phosphate-buffered saline (PBS).
